# Competitive motivation increased home use and improved prosthesis self-perception after Cybathlon 2020 for neuromusculoskeletal prosthesis user

**DOI:** 10.1186/s12984-022-01024-4

**Published:** 2022-05-16

**Authors:** Eric J. Earley, Jan Zbinden, Maria Munoz-Novoa, Enzo Mastinu, Andrew Smiles, Max Ortiz-Catalan

**Affiliations:** 1 Center for Bionics and Pain Research, Mölndal, Sweden; 2 Department of Electrical Engineering, Chalmers University of Technology, Gothenburg, Sweden; 3 Department of Clinical Neuroscience, Institute of Neuroscience and Physiology, Sahlgrenska Academy, University of Gothenburg, Gothenburg, Sweden; 4 Waterloo Engineering Bionics Lab, University of Waterloo, Waterloo, Canada; 5 Operational Area 3, Sahlgrenska University Hospital, Gothenburg, Sweden; 6 Department of Orthopedics, Sahlgrenska Academy, University of Gothenburg, Gothenburg, Sweden

**Keywords:** Cybathlon 2020, Neuromusculoskeletal prosthesis, Prosthetic control, Bionic arm, Osseointegration, Home use, Prosthesis training

## Abstract

**Background:**

Assistive technologies, such as arm prostheses, are intended to improve the quality of life of individuals with physical disabilities. However, certain training and learning is usually required from the user to make these technologies more effective. Moreover, some people can be encouraged to train more through competitive motivation.

**Methods:**

In this study, we investigated if the training for and participation in a competitive event (Cybathlon 2020) could promote behavioral changes in an individual with upper limb amputation (the pilot). We defined behavioral changes as the active time while his prosthesis was actuated, ratio of opposing and simultaneous movements, and the pilot’s ability to finely modulate his movement speeds. The investigation was based on extensive home-use data from the period before, during and after the Cybathlon 2020 competition.

**Results:**

Relevant behavioral changes were found from both quantitative and qualitative analyses. The pilot’s home use of his prosthesis nearly doubled in the period before the Cybathlon, and remained 66% higher than baseline after the competition. Moreover, he improved his speed modulation when controlling his prosthesis, and he learned and routinely operated new movements in the prosthesis (wrist rotation) at home. Additionally, as confirmed by semi-structured interviews, his self-perception of the prosthetic arm and its functionality also improved.

**Conclusions:**

An event like the Cybathlon may indeed promote behavioral changes in how competitive individuals with amputation use their prostheses. Provided that the prosthesis is suitable in terms of form and function for both competition and at-home daily use, daily activities can become opportunities for training, which in turn can improve prosthesis function and create further opportunities for daily use. Moreover, these changes appeared to remain even well after the event, albeit relevant only for individuals who continue using the technology employed in the competition.

**Supplementary Information:**

The online version contains supplementary material available at 10.1186/s12984-022-01024-4.

## Introduction

The loss of an arm is a traumatic experience that is usually followed by significant psychological and rehabilitation challenges. The interaction between engineering and science has, for a long time, made efforts to restore the functionality of a lost arm [1]. Nowadays, commercially available prostheses available for individuals with upper limb amputation can be electrically powered and operated via myoelectric signals, or body-powered and operated via shoulder movements on the contralateral limb. For those myoelectrically operated prostheses, the human–machine interface usually relies on electromyography (EMG) sensors placed on the surface of the stump. Myoelectric signals acquired from these sensors can be used to control the hand prosthesis via a conventional simplistic approach (direct control, or one-muscle one-movement) or via emerging advanced control schemes (*e.g.*, pattern recognition, musculoskeletal models, etc.), mostly explored in literature [2–4].

However, regardless to the hardware or control scheme in use, the dexterity and functionality of hand prostheses are still far from being comparable to a biological limb. High expectations of prosthetic hands are usually failed when presented with the reality of the challenges of skin-surface myoelectric acquisition (*e.g.*, electromagnetic noise, motion artifacts, impedance changes due to environmental conditions) and suspended socket attachments (*e.g.,* skin compression and abrasion, swell, and smell). Rejection rates as high as 40% have been reported for these devices [4]. Technologies including osseointegration and implantable sensors can address many of these interface challenges [5, 6], however the reality of prosthetic hands that function indistinguishably from biological limbs is still a ways off. Sadly, the distorted perception of current prosthetic technologies is quite often inflated by media.

In 2016, the Cybathlon was proposed with the mission of improving visibility and public understanding of physical disabilities and assistive technologies, focusing on six research disciplines including Prosthetic Arms [7]. Moreover, its competition format would drive research teams around the world to explore the needs of their users, develop and challenge new solutions, and improve the state-of-the-art in assistive technology. Indeed, retrospective manuscripts from the Cybathlon 2016 showcase many such developments from an engineering perspective [8, 9]. The perspectives of engineers and technology development play a big role at the Cybathlon, where many unique solutions have been hosted over the first (Cybathlon 2016) and second editions (Cybathlon 2020). However, these unique solutions also require some amount of practice, learning, and adaptation from the end user [10].

To facilitate this learning process, occupational rehabilitation and therapy are commonly adopted for assistive technologies, sometimes further augmented with serious gaming approaches [11, 12]. However, different people are motivated by different factors, such as competition or cooperative gaming [13]. For instance, competitive rehabilitation games have shown an increase in exercise intensity [14]. So, is it possible that a competition-based event like the Cybathlon might promote long-lasting behavioral changes in a prosthesis user motivated by competition?

In this study, we aimed to answer this question by investigating changes in prosthesis use for the pilot of the “x-OPRA” team before, during, and after the Cybathlon 2020. Specifically, we investigate these use changes in terms of active time while the prosthesis was actuated, ratio of opposing and simultaneous movements, and the pilot’s ability to finely modulate the speed of movements. Importantly, the investigation largely included home-use data logged on-board the prosthesis, which recorded information about how the pilot controlled his prosthesis in a manner similar to previous studies [15–18], possible because the pilot used the same prosthesis during the competition as he did at home. We show that the pilot’s use of the prosthesis at home increased even following the Cybathlon, compared to before his training period, and that his self-perception of his prosthetic arm and functionality has improved.

## Materials and methods

### The pilot

The research participant of this study, hereinafter defined as “the pilot”, was a 53-year-old male with left transhumeral amputation, acquired July 2015. He was implanted with a neuromusculoskeletal arm prosthesis in December 2018 following similar medical procedure as the patients reported in the work from Ortiz-Catalan et al*.* [6]. His neuromusculoskeletal arm interface (e-OPRA Implant System, Integrum AB, Sweden) consisted of:

- an osseointegrated percutaneous titanium implant system for direct skeletal attachment of the artificial limb,
- feedthrough connectors embedded in the osseointegrated implant to allow the artificial limb to communicate with implanted electrodes,
- 12 implanted intramuscular and epimysial electrodes on muscular target sites meant for myoelectric prosthesis control, and
- a spiral cuff electrode wrapped around the median nerve meant for somatosensory feedback purposes (not used in this study).

Moreover, the pilot underwent nerve transfers to native and free muscle grafts pursuing intuitive signal sources for myoelectric control. Details regarding the surgical intervention, the development of the reconstructed myoelectric sources, and the sites of the implanted electrodes are reported in [19].

This study was approved by the Swedish regional ethical committee in Gothenburg (Dnr: 769-12) and the pilot provided written informed consent.

### Prosthesis setup and daily usage monitoring

**Fig. 1:**
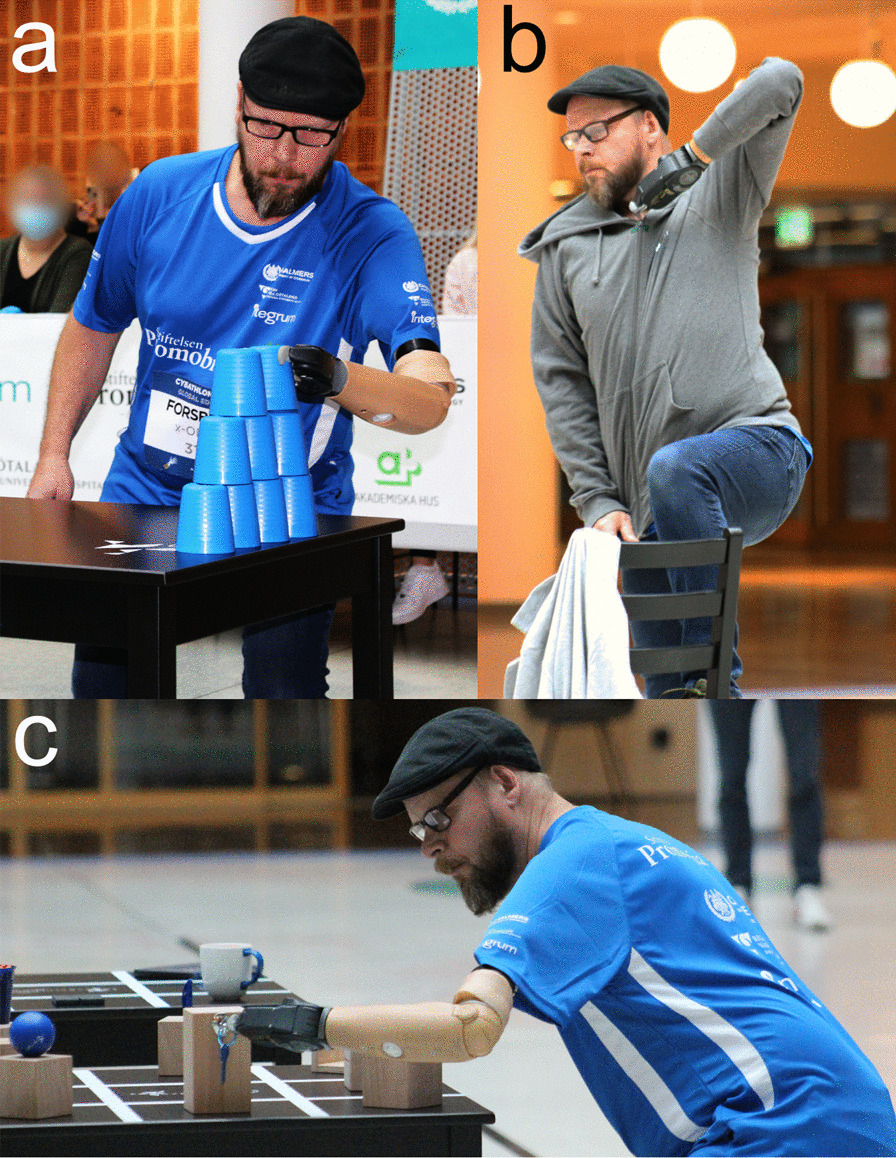
The x-OPRA team pilot with a left-side transhumeral amputation. The pilot used a Greifer terminal device, a wrist rotator, and an elbow with myoelectric lock/unlock both at Cybathlon 2020 and at home. When at home during *Cybathlon training*, the pilot reported spending a lot of time practicing with the cup stacking (**a**) and using his prosthesis to complete everyday tasks like donning and doffing clothing (**b**) and picking up everyday objects like keys (**c**)

The prosthesis was mechanically attached to the pilot’s stump via a clamp mechanism over the percutaneous portion of the osseointegrated implant. The myoelectric prosthesis setup comprised a Greifer prosthetic hand (Ottobock, Germany), a 12K50 elbow with controllable lock/unlock of the joint (Ottobock, Germany), and the Artificial Limb Controller (ALC, Integrum AB, Sweden) (Fig. 1), a custom-designed embedded system meant as the control interface between the neuromusculoskeletal interface and the prosthesis [20]. The Greifer hand was controlled via direct control strategy, consisting of the direct mapping of the prosthesis opening and closing speed (*i.e*., proportional speed control) to the mean absolute value of its corresponding myoelectric channel (lateral head triceps and reinnervated short head biceps, respectively). The mean absolute value was calculated from 100 ms non-overlapping windows of band-pass and notch filtered myoelectric data sampled at 500 Hz. At the start of training for the Cybathlon, the prosthesis setup was upgraded with a 10S17 wrist rotator (Ottobock, Germany), and the control algorithm was modified to permit simultaneous control of the wrist and hand. The wrist and the elbow were not controlled proportionally in speed, but instead with simpler on/off thresholds (pronation via median nerve RPNI, supination via reinnervated long head triceps, and elbow lock/unlock via long head biceps). All control settings, including EMG signal thresholds for prosthesis movement and speed range for each degree of freedom, were customized according to direct feedback from the pilot during visits to the lab.

Many studies tracking at-home prosthesis use require additional sensors such as accelerometers or pressure sensors [21]. However, the ALC includes a real-time logger which automatically stored data related to the prosthesis functioning in the on-board SD card [15]. Because the prosthesis that the pilot used during the competition was the same as his at-home prosthesis, we were able to track his use of the prosthesis throughout and after his training period.

While the prosthesis is powered on, the logger stores 10 data sets per second, appending these to the previously-logged data on the SD card. The logger does not have the capability to label each data point with a precise time and date (there is no on-board real-time clock), however it includes a flag variable signaling every system reboot whenever it is powered on. Based on these reboot flags and the storing frequency of the logger, it was possible to estimate the lengths of time the prosthesis was powered on, hereafter defined as a “session”, by counting the number of data sets saved between two reboot flags. Each data set included predicted movement, predicted speed (if relevant), and inertial sensor data.

The real-time data logger collected data from 17 December 2019 to 3 March 2021, broken up into three separate datalogs summarized in Table 1. These datalogs correspond to before, during, and after the pilot’s training for the Cybathlon competition held on 13 November, 2020 [22]. Such data represents the entire prosthesis usage of the pilot in that period, regardless of the context where the prosthesis was used (*e.g.*, in lab or at home).

**Table 1:** Datalog summary

Phase	Starting date	Ending date	Phase duration	Powered wrist	Maximum battery life	Average daily use
Pre-Cybathlon	17 December 2019	7 July2020	203 days	No	21 h	10.98 h
Cybathlon Training	31 August 2020	14 November 2020	74 days	Yes	6 h	5.28 h
Post-Cybathlon	14 November 2020	3 March 2021	110 days	Yes	13 h	11.34 h

Average daily wear is estimated by normalizing the phase’s datalog duration by the total phase duration

### Training protocol

The pilot for the “x-OPRA” team began training after receiving a powered wrist on 31 August 2020. The training schedule was ideated at the Center for Bionics and Pain Research, Sweden, and consisted mainly of a structured repetition of all major tasks included in the Cybathlon Powered Arm Prosthesis discipline, enhanced with strategy and performance suggestions from the research team [23]. For this endeavor, a replica of the circuit was built and located in a common area of the Chalmers University of Technology, Johanneberg campus, Gothenburg, Sweden. The training schedule aimed to have the pilot exercising the ARM tasks for a full day training session every lab visit. An in-lab training session included practicing the individual Cybathlon ARM tasks, fitting sessions to iteratively optimize the control settings according to direct feedback from the pilot, team strategy discussions, and periodic breaks. During some of these training sessions, our other pilot (from the “e-OPRA” team) also trained, and the two pilots would collaborate and learn techniques from one another.

The more substantial component of the pilot’s Cybathlon training was conducted at home using a variety of techniques and training materials. The pilot reported spending a lot of time practicing with the “cup stacking” task requiring him to stack 10 plastic cups in a pyramid as quickly and precisely as possible (Fig. 1a). Finally, the pilot reported that he sought out new ways to use his prosthesis in daily life as a means of training. Some of these tasks were also included in the Cybathlon (such as tying shoelaces, donning and doffing clothing (Fig. 1b), and picking up everyday objects like keys with the prosthesis (Fig. 1c), while others were more spontaneous and improvisational (such as washing dishes or performing car repairs).

### Data analysis

The quantitative analysis performed in this study was based on the data stored from the real-time data logger. All analyses are post-hoc, meaning that none were planned before the data were collected and analyzed. This analysis primarily aimed to investigate differences in the pilot’s prosthesis use and control before, during and after his training for the Cybathlon competition. For this purpose, only data regarding movement and speed predicted were used. The data were checked and confirmed to be free from spurious instances and errors. Only datalogs longer than 5 min in total length were included in analyses. For data averaged across the duration of each phase, statistical analyses were not possible due to the lack of on-board real-time clock. For data presented longitudinally over each phase, median and quartiles are presented for summary, and differences across phases were determined using the Wilcoxon rank sum test. Statistical comparisons of similarity between distributions were performed using two-sample Kolmogorov–Smirnov tests.

### Daily active use

Analysis of the daily active use sought to determine if the pilot used the prosthesis more actively following the Cybathlon. Daily active use was defined for each phase (*pre*-, *training*, and *post-Cybathlon*) as the average daily use (estimated by normalizing the phase’s datalog duration by the total phase duration) multiplied by active prosthesis use rate (calculated as the ratio between the time while the prosthesis was performing active movements and the total time the prosthesis was turned on). Daily active use was calculated separately for the hand, wrist, and elbow degrees-of-freedom. It should also be noted that daily use and daily active use are not the same as daily wear – the ALC datalogger only records data while the prosthesis is powered on, thus time that the prosthesis is worn but powered off (*e.g.,* if grasping objects for a long time with the prosthesis powered off) is not included.

### Prosthesis symmetry and simultaneity

Symmetry and simultaneity are used to describe the relative ratio of opposing movements for the same degree of freedom (symmetry) and the proportion of movements which were performed simultaneously with movements from another degree of freedom (simultaneity). Each movement is plotted as the ratio of the number of movement commands to the total movements for that degree of freedom; thus, for a single degree of freedom, the ratios of both movements add up to 100%. This rate was calculated for each session and separated between the hand and wrist degrees of freedom. Symmetry/asymmetry were calculated as the difference in percentages between opposing movements for the same degree of freedom, and thus ranged from -100% to + 100%, with 0% indicating perfect symmetry. This analysis was intended to identify pilot-preferred movements. Due to frequent misclassifications where the wrist was activated during hand closing, the simultaneous wrist and hand closing functionality was turned off.

### Hand open/close proportionality

Analysis of the hand open/close proportionality sought to investigate changes in the pilot’s ability to finely modulate the speed of the hand prosthesis. The proportional speed at each control decision was calculated as an integer percentage of the pilot’s myoelectric range defined during fitting; furthermore, the minimum and maximum prosthesis speeds were tuned according to pilot preference. The distribution of these percentages for both hand open and hand close are calculated for each session. This analysis was intended to investigate whether the pilot developed a more accurate and versatile control of all possible speed levels available on the prosthesis, as opposed to a more simplistic on/off approach. Thus, a more uniform or variable range of prosthesis speeds is considered to indicate a higher capacity for myoelectric control. Two-sample Kolmogorov–Smirnov tests were used to determine if two distributions of movement speeds (normalized by the permitted speed range) differed.

### Qualitative data

During Cybathlon training, the team routinely performed informal and unstructured telephone interview follow-ups with the pilot every other week. The purpose of these follow-ups was to track his prosthesis control, Cybathlon training, and perception of his prosthesis at home, as well as to identify potential issues with or improvements to the prosthesis or control that could be addressed during his next lab visit.

Moreover, a semi-structured interview was conducted with the pilot in September 2021 (six months after the end of the *post-Cybathlon* phase) to corroborate analytical findings from the on-board datalog and to obtain his retrospective perception of the Cybathlon experience, especially to comprehend the effect the competition had on his prosthesis use and perception after the event. The pilot was asked about how he used his prosthesis for daily tasks and if there were activities he felt unable to do prior to training for the Cybathlon.

## Results

### Daily active use

Table 1 shows an overview of the three datalog phases: *pre-Cybathlon*, *Cybathlon training*, and *post-Cybathlon*. The pilot used his prosthesis an average of 10.98 h per day before the Cybathlon, 5.28 h per day during the training period, and 11.34 h per day after the Cybathlon. Furthermore, the battery life dropped from 21 h (*pre-Cybathlon*) to only 6 h after installing the wrist rotator (*Cybathlon training*); because the battery was installed inside the arm it could not be swapped out, the pilot had to doff the prosthesis to charge it. This battery life issue was only fixed after the Cybathlon by fitting the prosthesis with a higher-capacity battery.

**Fig. 2:**
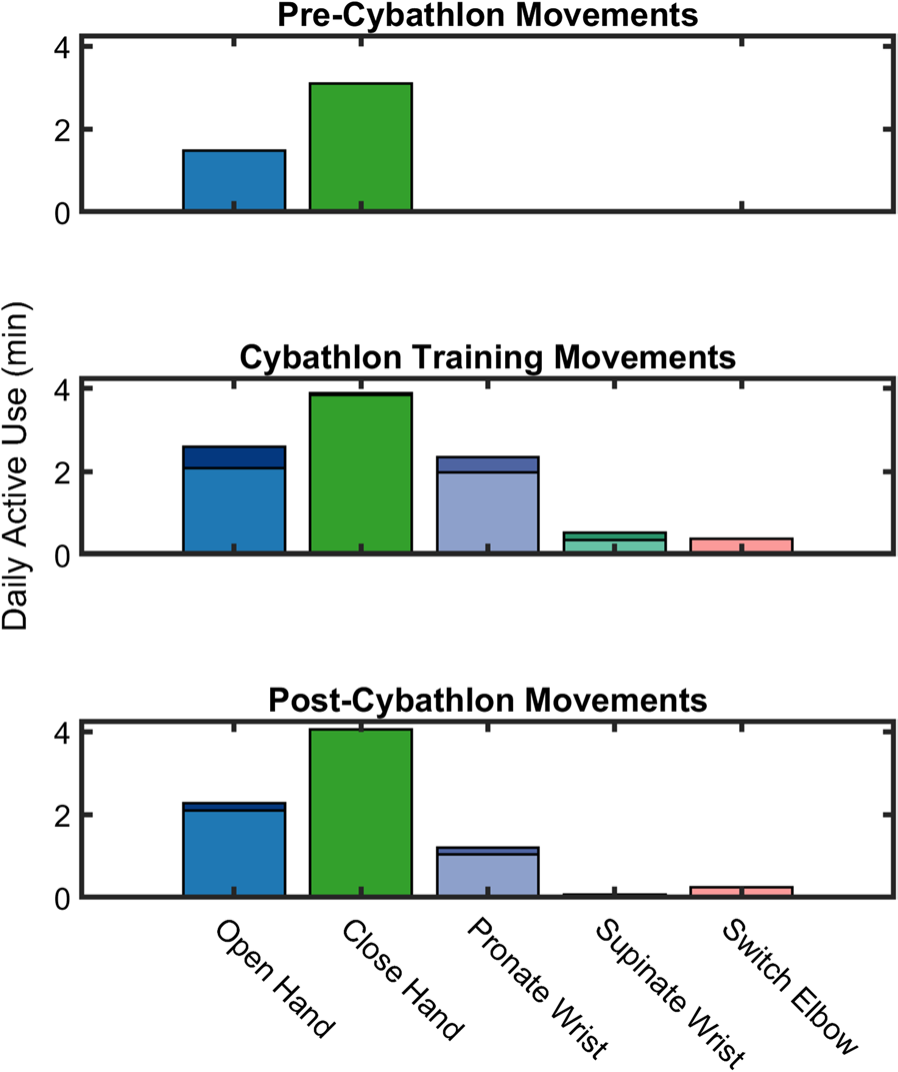
Daily active use increased during *Cybathlon training* and *post-Cybathlon*, compared to *pre-Cybathlon*. Bars show the aggregate rate of predictions during the three phases, with simultaneous control predictions shown as the darker stacked bars. It should be noted that simultaneous control was not possible while closing the hand, a decision made to improve prosthesis control for the pilot

Figure 2 shows the average daily active use during each phase. Because powered wrist rotation was only added at the start of the *Cybathlon training* phase, there was no wrist pronation or supination during the *pre-Cybathlon* phase, and no 2 degree of freedom movements.

The average daily use of the prosthetic hand (opening and closing) increased by 42% from 4.59 min *pre-Cybathlon* to 6.52 min during *Cybathlon training*. The elbow was also used 8.5 times more during *Cybathlon training* (0.38 min) than *pre-Cybathlon* (0.04 min). The wrist, which was a new addition for the pilot, was also used frequently during *Cybathlon training* (2.88 min). During *Cybathlon training*, 8.46% of hand movements were performed simultaneously with the wrist.

In the *post-Cybathlon* phase, daily active use had dropped to 7.67 min – lower than during *Cybathlon training*, but still 65.7% higher than *pre-Cybathlon*. Of note, the hand use (6.35 min) was about the same as during *Cybathlon training*, though the wrist (1.27 min) and elbow (0.25 min) use were lower. The rate of simultaneity (2.91%) also dropped after *Cybathlon training*.

It should be noted that the daily active use only accounts for active movement of the prosthesis and does not account for passive use such as holding objects with a steady force. Further, to put the daily active use into perspective, the prosthetic hand is capable of fully opening within 200 ms, meaning that even a few seconds of active use could easily incorporate numerous grasping actions. Similarly, locking or unlocking the elbow also takes about 200 ms.

**Fig. 3:**
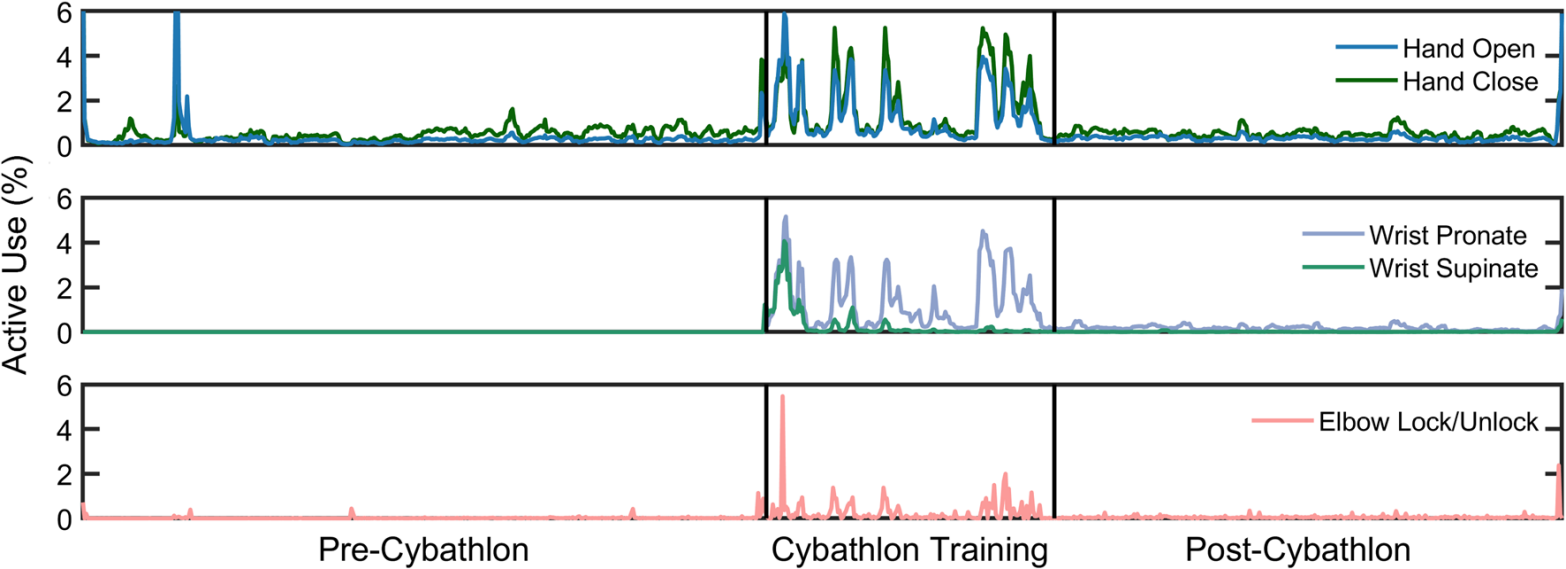
Active use rates of the prosthesis increased dramatically during *Cybathlon training*, and *post-Cybathlon* active use rates were higher than those *pre-Cybathlon*. Active use is defined as the percentage of time during prosthesis use that a given movement is being performed, as opposed to another movement or rest. Plotted values are a moving average of 5 datalog sessions, to improve readability. Vertical black bars indicate the breaks between the *pre-Cybathlon*, *Cybathlon training*, and *post-Cybathlon* datalogs. Powered wrist pronation and supination were only added at the start of *Cybathlon training*

The longitudinal active use rates of each degree of freedom are shown in full detail in Fig. 3, depicting how active use changed over time. The changes in active use rate shown in Fig. 2 are also apparent here, with active hand use increasing from *pre-Cybathlon* (median [quartiles]: 0.70% [0.39%, 1.16%]) to *Cybathlon training* (1.95% [0.88%, 5.84%], *p* < 0.001). Active hand use also increased *post-Cybathlon*, compared to *pre-Cybathlon*, though not by a significant margin (0.78% [0.51%, 1.11%], *p* = 0.142). Active elbow use followed a similar trend, with the lowest rated *pre-Cybathlon* (0.005% [0.0005%, 0.013%]), and significantly higher during both *Cybathlon training* (0.086% [0.024%, 0.339%], *p* < 0.001) and *post-Cybathlon* (0.032% [0.019%, 0.055%], *p* < 0.001). However, contrary to our expectations, there was no gradual increase in active use rate over the course of the *Cybathlon training* period; instead, active use seemingly spiked and remained constant throughout the period.

One other behavior apparent from Figs. 2 and 3 is the preference of hand closure over hand opening, and of wrist pronation over wrist supination. The hand behavior is also observed in our previous study [17]. The wrist behavior is explored further in the following section.

### Prosthesis symmetry and simultaneity

**Fig. 4:**
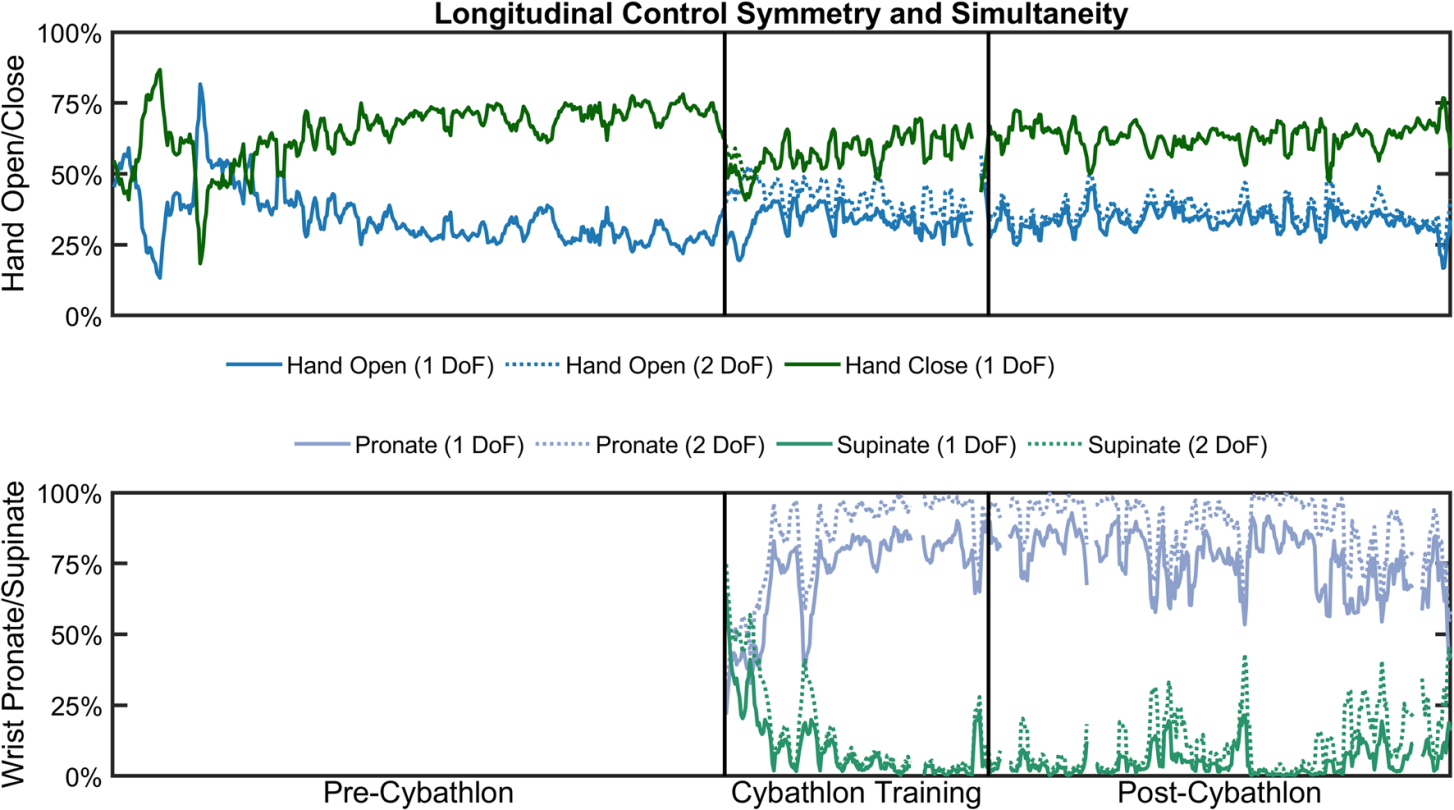
Control symmetry of the prosthetic hand remained about constant though all phases, however prosthetic wrist movements started symmetric before shifting towards more frequent pronation. Powered wrist pronation and supination were only added at the start of the *Cybathlon training* phase, and control simultaneity was higher during *Cybathlon training* than *post-Cybathlon*. Plotted values are a moving average of 5 datalog sessions, to improve readability. Solid lines show the proportion of single degree of freedom commands for each given movement, while dotted lines show the additional two degree of freedom commands for the movement. Vertical black bars indicate the breaks between the *pre-Cybathlon*, *Cybathlon training*, and *post-Cybathlon* datalogs

Figure 4 shows the symmetry of the prosthetic hand and wrist before, during, and after the Cybathlon, with the simultaneity denoted on top with dashed lines. The top plot reveals a slightly higher rate of hand close over hand open. This is to be expected if, for example, the pilot is closing the hand more slowly and therefore taking a longer time (*i.e.*, more *hand close* commands) to complete the task. However, the median asymmetry *pre-Cybathlon* (36.04% [19.90%, 46.91%]) became more symmetric during *Cybathlon training* (18.49% [8.35%, 26.49%]; *p* < 0.001). Although hand asymmetry increased again *post-Cybathlon* (28.33% [20.49%, 33.69%], *p* < 0.001), it remained more symmetrical than *pre-Cybathlon* (*p* < 0.001).

The preference for wrist pronation noted in Figs. 2 and 3 is also shown here. The pilot started *Cybathlon training* with a balanced ratio of wrist pronation to wrist supination. However, after a few sessions, he began to develop a pattern of relying primarily on wrist pronation, likely due to the ease with which he was able to perform the movement. By the *post-Cybathlon* phase, the median wrist asymmetry (91.51% [74.50%, 98.48%]) was slightly higher than during *Cybathlon training* (87.29% [54.41%, 96.63%], *p* = 0.026).

### Hand open/close proportionality

**Fig. 5:**
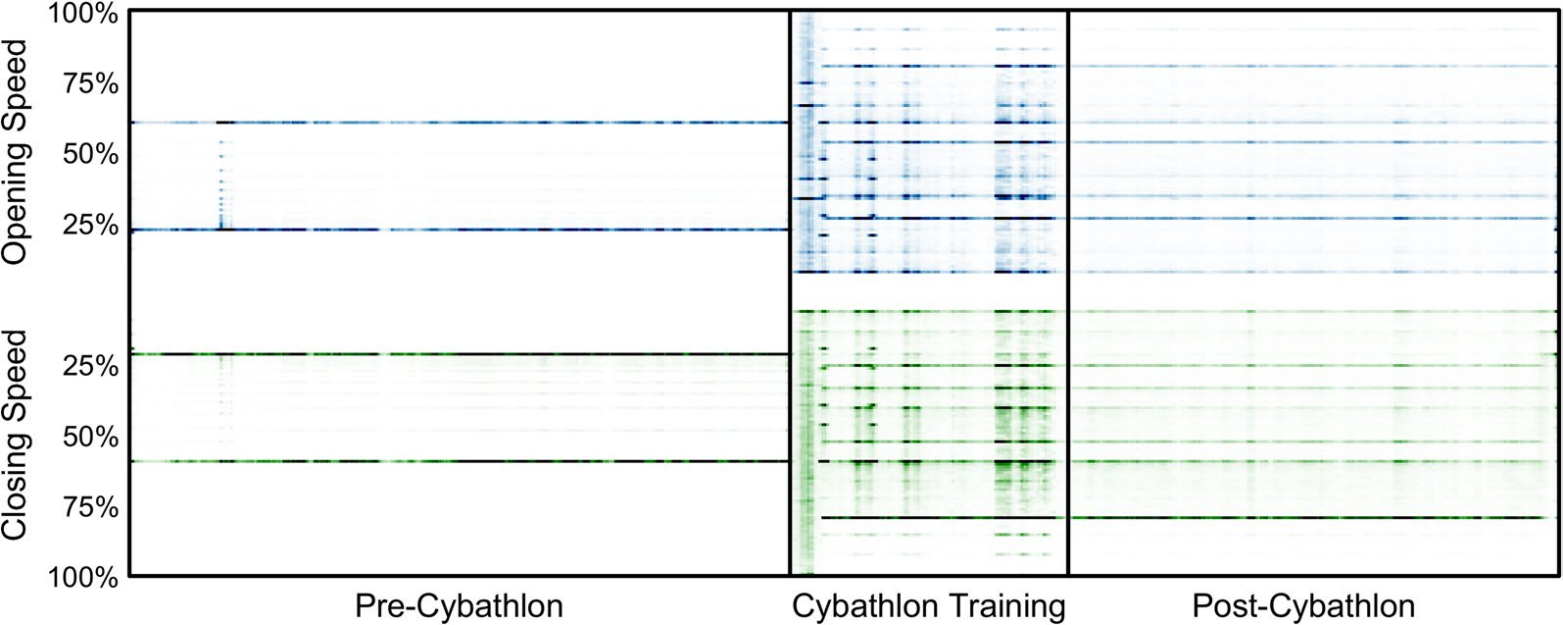
Prosthetic hand speeds were primarily bimodal *pre-Cybathlon*, but the pilot started using a more uniform distribution of possible speeds during and after *Cybathlon training*. Plotted values are a moving average of 5 datalog sessions, to improve readability. The intensity of blue and green points indicates the relative prevalence of different proportional levels of hand open and hand close, respectively

Figure 5 shows how the distribution of hand movement speeds changed between *pre-Cybathlon*, *Cybathlon training*, and *post-Cybathlon* phases. Each column of colored points represents the distribution of discrete proportional hand speeds achieved in one session, with more intense shading indicating the relative prevalence of hand speeds in the given session. Prior to the start of *Cybathlon training*, the pilot essentially used only two speeds for each movement: a slow and a fast speed. However, after upgrading the prosthesis for the Cybathlon and tuning the movement thresholds and motor speeds to his preference, the pilot started using a wider range of possible speeds including both slower and faster speeds, as well as a more uniform distribution of speeds between the extrema (*p* < 0.001). Furthermore, this changed behavior was retained after the conclusion of the Cybathlon.

### Qualitative data

Throughout this study, frequent and short follow-ups were conducted over the phone. Furthermore, a semi-structured retrospective interview was conducted in September 2021 (six months after the end of the *post-Cybathlon* phase) at the Center for Bionics and Pain Research [see Additional file 1]. Both the follow-ups and the semi-structured interview provided valuable information about the pilot’s perception of his prosthesis.

Before training for the Cybathlon, the pilot considered his prosthetic control to be suboptimal. He presented issues locking and unlocking the elbow, and after being fit with wrist rotation at the start of *Cybathlon training*, the pilot noted that it required a lot of attention to control the wrist. However, the pilot highlighted that the Cybathlon drove him to use his prosthesis more and in new ways such as opening doors, cooking, washing the dishes, and performing car maintenance and repairs. While experimenting with using his prosthesis, he also found that he performed more bilateral movements, and that even after the competition had ended, he found himself using his prosthesis more than before. Some of the tasks he used to train for the Cybathlon, like tying his shoes, were things that he was unable to do himself before training, but that he now continues to do to this day, allowing him to live more independently.

The pilot’s perception of his prosthesis also changed over the course of *Cybathlon training*. Before the competition, he felt his prosthesis was more of a tool. In the time since the Cybathlon, though, he now feels that his prosthesis is more a part of his body, commenting, “When I take my prosthesis off, I feel like something is missing.” The pilot also noted that his movements felt more natural, and that what he likes most about his prosthesis is that he now trusts it more.

Overall, in the pilot’s opinion, the main impact that the Cybathlon had on him was an increase in self-confidence, both in terms of the control of his prosthesis and in being in front of a camera. Furthermore, even within the competitive atmosphere of the competition, one of his highlights was learning from, practicing with, and becoming friends with the e-OPRA pilot, and he is hoping to participate again in the next Cybathlon edition.

## Discussion

### Insights from the datalog

In this study, we investigated how training for and participating in a competitive event like the Cybathlon affects the active use time of a myoelectrically-operated prostheses, the pilots’ preferences of performed movements, and the pilot’s ability to finely modulate the speed of movements.

Indeed, the pilot reports that the Cybathlon acted as a catalyst to increase his daily prosthesis use. Especially during the active training for the Cybathlon, which included both in-lab and at-home training sessions, his active prosthesis use had doubled, and he executed movements with significantly more granularity. Training for the Cybathlon also promoted the direct incorporation of the additional wrist degree of freedom the pilot was provided with in preparation for the competition. Of note is that 14.6% of *post-Cybathlon* wrist movements were performed while opening the hand. Although the datalog, in isolation, is unable to differentiate between volitional and nonvolitional simultaneous control, conversation with the pilot suggests this movement is volitional, if subconscious. These types of movements would most often be associated with prepositioning of the hand during reach-and-grasp tasks, which suggests that the pilot was utilizing the simultaneous functionality of the prosthesis to fluidly complete daily tasks. This fluidity may not have been as easily achieved with sequential control techniques available for many commercially available prostheses, and the continued use of this simultaneity after the Cybathlon demonstrates the utility and benefit of this control.

Moreover, the pilot continued to use his prosthesis more actively and wore it for longer even after the Cybathlon competition compared to his *pre-Cybathlon* use. The decrease in use compared to the *Cybathlon training* phase emphasizes that continuous motivation, *e.g.*, in form of competition-based events or competitive rehabilitation games [14], can have an advantageous effect on learning and corroborate the use of assistive devices. This study has been limited to one subject, but it is not unlikely that other individuals as motivated as our pilot would show similar increases in prosthetic use.

Daily use (Table 1) and daily active use (Fig. 2) values are averaged over the course of at least a couple of months, giving some confidence to the calculated durations. However, it should be noted that the pilot’s daily use of the prosthesis may not necessarily be representative of users of the same prosthetic system, as participants in our previous study showed daily use of up to 18 h per day [17]. Similar studies with different prosthetic systems have also reported average daily use of 4–8 h per day [16, 18]. These differences in daily use could arise from multiple sources, including differences in job requirements, hobbies, battery life of the prostheses, and others, and identifying these differences is outside the scope of this study.

In general, the a posteriori analysis of home-use data can be an important tool to further improve the standard in prosthetic control. Suboptimal control parameter settings that lead to *e.g.*, disuse of a prosthetic movement like we observed with supination, can now be easily spotted by the engineers. This in turn can improve the prosthetic fitting process and have a long-term effect on how a prosthesis is used during daily life.

### Comparison between datalog and pilot conception

To supplement our findings from the datalog, we conducted a semi-structured interview with the pilot to ask about his use and perception of his prosthesis before, while training for, and after the Cybathlon. In this interview, the pilot claimed that training for the Cybathlon gave him motivation to use his prosthesis more, and in ways he had not done so previously; indeed, this increased use is evident in the doubling of daily active use shown in Fig. 2, and the spike in active use rate shown in Fig. 3. Before analyzing the data, we had expected the active use rate to gradually increase over the course of the *Cybathlon training* period, plateauing in the sessions nearing the Cybathlon. Contrary to our expectations, however, active use seemingly spiked and remained constant throughout the period, which may be due in part to the fact that he used the same prosthesis at home as he did during the competition. This allowed him to turn even everyday tasks into training opportunities, and he challenged himself to use the prosthesis even when he normally would not. This increased prosthesis use, in turn, helped the pilot to feel less handicapped, able to do more things with his prosthesis instead of his intact hand.

At the beginning of the *Cybathlon training* period, we enabled simultaneous control of the prosthetic hand and wrist, whereas previously the wrist had to be rotated manually with the intact hand. During the follow-up calls and the interview, the pilot expressed his satisfaction with the rotating wrist, saying that although it did not enable him to do anything he could not do before, it did become easier to preposition his hand when reaching to grab objects. This can again be seen from the wrist activity in Fig. 3. Furthermore, his use of 2 degree of freedom movement as shown in Fig. 2 suggests that the pilot will sometimes rotate the wrist and open the hand simultaneously. While the pilot did not recognize it, his wife (who was present during the interview) had noted that he was doing these coordinated movements more often while reaching for objects, which may suggest that these types of coordinated movements have become somewhat second-nature to the pilot. One behavior which was recognized by the pilot was his preference of wrist pronation over wrist supination. Although his uses of both wrist rotation directions started somewhat balanced at the beginning of *Cybathlon training*, over time he found pronation easier to control and learned to rely on pronation any time he needed to position the wrist. This is clearly visible in Fig. 4, where the wrist symmetry greatly skews in favor of wrist pronation during the *Cybathlon training*. When asked about his control of the speed of the prosthetic hand, the pilot did not recognize any difference in his use of fast and slow speeds. Although this contradicts the change in speed distribution from bimodal *pre-Cybathlon*, to uniform during *Cybathlon training* (Fig. 5), this may be due to a couple of factors. First, it is possible that the pilot is not paying attention to the speed of the hand, or that the visual or auditory differences in speed are difficult to differentiate due to perceptive uncertainty [24]. Second, a linear change in speed, as controlled by the ALC, may not relate to a linear increase in the speed of the prosthesis, making these differences in control signals further difficult to discern. It is possible that providing sensory feedback of the speed calculated from the control signals may help the pilot to better identify his prosthesis speed, or to adapt to changing conditions [25].

Perhaps the most impactful change for the pilot is that, after having pushed himself to use his prosthesis more in preparation of the Cybathlon, he now views the prosthesis more as a part of himself. During the semi-structured interview, the pilot offered, “I don’t feel like I have one arm anymore. Now it feels more natural.” When asked to elaborate, he explained that while the practice in the lab was helpful, his home use and training with the prosthesis played a bigger part in his improvement in control, and likely his perception of his prosthesis. This perception is in line with previous findings on individuals who have gained higher function and increased prosthetic use with a neuromusculoskeletal prosthesis [26].

Overall, the information from the datalogs and the pilot interviews suggest that participating in the Cybathlon competition gave him the motivation to use his prosthesis more at home. As a result, he continues to use his prosthesis more in daily life, which has improved his perception of his prosthesis and his self-confidence. Commenting on his Cybathlon experience as a whole, the pilot said, “The Cybathlon helped me to use my prosthesis more in daily life. I think that was good for me. That was my reason for doing the race.”

## Limitations

The pilot’s neuromusculoskeletal prosthetic system was primarily designed with translating prosthetic technology to reliable home-use in mind [6]. Competing in an event like the Cybathlon, which focuses on activities of daily living as the central tasks, provides a unique avenue by which training for the event can directly impact at-home functionality of prosthetic arm users. From this perspective, the self-contained nature of the e-OPRA system is perfectly situated to this type of competition. However, this same self-contained nature also limits the amount of data that can be collected while outside of the lab. The pilot did not wear external sensors [21]; instead, we relied on the periodic logging of ALC control variables and on-board sensors [15]. This type of analysis permits longitudinal analysis of prosthesis use, however interpretation of these datalogs in a real-world context necessitates consideration of the limitations of the logged data.

Many of the logged variables are consistent with other at-home prosthesis use studies, including the on-board wear time and active use rates of different movements [18]. However, because our pilot’s prosthesis did not have a pressure sensor, we were not able to log object interaction, which has previously been used to provide additional context for home use [16]. Furthermore, the lack of an on-board real-time clock prevented us from performing any analyses related to day-to-day or time-of-day behavior [27].

Daily active use was estimated by dividing the total time logged during each phase (*pre-Cybathlon*, *Cybathlon training*, and *post-Cybathlon*) by the total duration of that phase (determined by start and end dates of each datalog). Daily active use only considers time that the prosthesis is powered on, therefore it is not the same as daily wear if the prosthesis is worn while being powered off. Similarly, daily active use only considers the time spent actively moving the prosthesis but does not consider passive uses of the prosthesis such as holding, carrying, or pushing objects. Accordingly, the prosthesis may be “used” more than what is presented in this study, but in ways that are not captured by the datalog and would require additional sensors or cameras.

We also wish to clearly state that the data and experiences presented in this study are only for a single pilot, and therefore the findings presented here may not necessarily generalize to all prosthesis users.

## Conclusions

A competition-based event like the Cybathlon can indeed promote behavioral changes in how people with amputation us their prostheses. Especially during the preparation leading up to the event, our pilot remarkably increased his active prosthetic use, carried out tasks with more granular control, and learned to perform new activities of daily living with his prosthesis. Even following the conclusion of the event, the pilot has had longitudinal improvements in his home prosthesis use and his self-perception of the prosthesis. He continues to perform the activities of daily living learned during the Cybathlon, granting him more independence in his daily life. It is worthy of notice that such benefits can only be attained if participants continue using their assistive technologies after such competition.

## Supplementary Information

**Additional file 1.** x-OPRA pilot interview. This file includes a transcript of the semi-structured retrospective interview conducted in September 2021, six months after the end of the *post-Cybathlon* phase.

## Data Availability

Data and materials produced during this study can be made available upon reasonable request.
