# Supplementary material for: Competitive motivation increased home use and improved prosthesis self-perception after Cybathlon 2020 for neuromusculoskeletal prosthesis user
Source: J Neuroeng Rehabil. 2022 May 16;19:47. doi: 10.1186/s12984-022-01024-4 (PMC9112467; doi:10.1186/s12984-022-01024-4)
Supplement: Supplementary file 1 — Additional file 1. x-OPRA pilot interview. This file includes a transcript of the semi-structured retrospective interview conducted in September 2021, six months after the end of the post-Cybathlon phase. [file 12984_2022_1024_MOESM1_ESM.pdf]

Semi-structured interview Cybathlon 2020 – “x-OPRA” pilot

I. Introduction

The *Researchers* performed the following semi-structured interview at the Center for Bionics and Pain Research (CBPR) laboratory, Gothenburg, Sweden, on the 6 of September 2021. The interview was video recorded under the authorization and consent of the *“x-OPRA” pilot*.

The interview started with the *Researcher* explaining to the *Pilot* the aim of the semi-structured interview. The aim was to obtain pilot’s perception about his experience at the Cybathlon competition 2020 and the impact it has had on his prosthesis use and his life.

Portions of the interview were conducted in Swedish and later translated to English for the purposes of this transcript. The transcript was also lightly edited for grammatical clarity and to remove personal details.

II. Questions:

Researcher

What is your opinion on the prosthesis that you used for the competition? What do you like about that hand? What do you not like about that hand?

Pilot

I trust it. It has good grip, and it’s fast.

Researcher

What is it about the Greifer that you put your trust in?

Pilot

I have a better grip. With the other hand, I drop things. This is stronger.

Researcher

Yeah, it's a very useful system, certainly. What would you say are the strengths of the system that you have? Not just the Greifer, but also the osseointegration, the control algorithms?

Pilot

The movements, I can trust them. Its feels like my own arm.

Researcher

How do you like the way that you control the prosthesis?

Pilot

It comes naturally. I just think.

Researcher

What would you say are the weaknesses or the drawbacks of the system?

Pilot

Before it was the battery. It is better now.

Researcher

Are there things that you feel that you cannot do with your prosthesis?

Pilot

No.

Researcher

You could do anything?

Pilot

Yes, almost. Yeah

Researcher

Before you were given a rotating wrist, what are some of the activities that you typically used your prosthesis for? So, for example, gardening, housework, cooking?

Pilot

Everything. Work, fixing cars, putting on clothes.

Researcher

Do you do housework? (To the pilot's wife) Does he do housework?

Pilot

(Laughter) Sometimes?

Pilot's Wife

Yes, sometimes.

Pilot

I do the dishes.

Researcher

How is your cooking?

Pilot

Mmm, not so good. (Laughter) But I made a fantastic lasagna last week.

Researcher

After we gave you the rotating wrist, which was around July of last year, are there any new activities that you are able to do because you have that wrist, that you couldn't do before?

Pilot

No. But it's easier to get a good position of my arm. Yeah, it helps a lot.

Researcher

Are there any activities that you stopped doing now that you have that wrist? Is there anything that you used to do, that you don't do anymore?

Pilot

No.

Researcher

Has the wrist been detrimental? Has it caused problems as you've used it?

Pilot

No.

Researcher

The wrist always responds as it's supposed to?

Pilot

Yeah.

Researcher

Okay. I'm happy to hear that.

Pilot

Sometimes, it would open when I supinate. But I knew it would happen, so it's no problem.

Researcher

If you're like positioning your hands to grab on to something, do you supinate and pronate about equally? Or do you have one that you prefer over the other?

Pilot

Maybe pronate more.

Researcher

And why is that?

Pilot

I think is easier to do that one. But it's not a big difference.

Researcher

When you were practicing for the Cybathlon, I know our dear friend Andrew was very impressed with the amount that you have been practicing and training for the events, like Rocky [Balboa] on the stairs. So, how did you train for the Cybathlon?

Pilot

At home, I trained with the cups, the scissors… Almost everything, I think.

Researcher

Did you feel that, while you were training for the Cybathlon, you used your prosthesis more often or less often?

Pilot

Yeah, more often. I pushed myself to use it more.

Researcher

What are some ways that you use it more outside of the training with the cups?

Pilot

Open doors, operating lights, eating. Tying my shoes.

Researcher

Are those things that you would normally do? Or did you do them because you're training?

Pilot

Before I was training, my wife always tied my shoes. But now, I'm a big boy and I do it myself. (laughter)

Researcher

No more Velcro for you. (laughter)

During that period while you were training for the Cybathlon, did you see any changes in how your control of the prosthesis has changed?

Pilot

Yeah, it was much better.

Researcher

Why do you think you got better? Do you think it was better because you were training and practicing?

Pilot

Yeah.

Researcher

Do you think it also improved because of changes that we made in the lab?

Pilot

Yeah, that too. Training and the changes.

Researcher

Which of those two do you think had a bigger impact on your control now?

Pilot

Training, I think.

Researcher

And when you say that your control improved, in what way would you say that it improved?

Pilot

I think my [control] signals were better when I was training.

Researcher

Did the hand react the way that you would expect it to more or less?

Pilot

Yes, more.

Researcher

You are using the prosthesis in a lot of different ways. Ways that you hadn't used it before. Did you try to use the prosthesis at different speeds and in different arm positions? Or did you feel that you basically use it the same way?

Pilot

The same way. But I use it more.

Researcher

How did the Cybathlon influence your perception of your prosthetic system, the way that you view it, the way that you feel about it? Do you like your prosthesis more now, having been part of the Cybathlon, or would you say that you feel about the same about it?

Pilot

More, I think.

Researcher

You said that while you were training you use the prosthesis more. Do you feel that you use the prosthesis more now than you did before the Cybathlon?

Pilot

Yes.

Researcher

Do you consider the prosthesis more a part of you, now?

Pilot

Yeah.

Researcher

Have you always considered the prosthesis to be a part of you?

Pilot

No. But after the Cybathlon, I felt it.

Researcher

How did you view it before?

Pilot

Like the prosthesis was not a part of my body.

Researcher

Even when you had the osseointegration, it was still a tool?

Pilot

Yeah.

Researcher

And what was it about the Cybathlon that helped you to view the prosthesis more as a part of you?

Pilot

Because I use it more and I forced myself to use it. Yeah, I think it was that.

Researcher

So, one of the things that we have been working on for a while now with your prosthesis, is not only giving you the ability to open and close and to rotate, but to do both of those at the same time. So, for example, to grab onto something and close the hand or close the hand and rotate. How often would you say that you use that functionality?

Pilot

Everyday. Although maybe I just grab something and then rotate.

Pilot's wife

No, but I saw you do that the other day.

Pilot

But I don't think about it.

Pilot's Wife

That's the point. Just in the last weeks, he been doing it like this (moves her arm in a wide arc while pronating her hand) to grab something. And it goes naturally.

Researcher

Could you demonstrate that?

Pilot's Wife

Like reaching for something like this (moves her arm in a wide arc while pronating her hand), and before it was like this (first moves her arm more slowly, and then pronates her hand after moving).

Pilot

I don't think about it.

Researcher

You don't think about it. Okay. Very interesting. The fact that you're doing that while your arm is moving as well, is very good.
A while back, since the Cybathlon had finished, would you say that you still used the prosthesis the same amount?

Pilot

Yeah, I think so.

Researcher

Did you continue training after the Cybathlon?

Pilot

After the Cybathlon, I maybe used my non-affected arm more. Before, I forced myself to use my prosthesis.

Researcher

Are there things that you continue doing now that the Cybathlon is over, that you haven't done before?

Pilot

Yeah, tying my shoes.

Researcher

Making lasagna. (laughter)

Pilot

Yeah. And I work more with my two hands.

Researcher

So, your prosthetic hand has the ability to open both slowly and quickly, and everywhere in between. How would you say that your control of that speed was before you started training for the Cybathlon?

Pilot

I think it was good.

Researcher

Do you think you used a lot of different speeds when opening and closing the hand?

Pilot

Yeah, sometimes.

Researcher

And do you think that carried over into the Cybathlon?

Pilot

I think is the same.

Researcher

And then after the Cybathlon, you think it is also still the same?

Pilot

Yeah.

Researcher

So, the speed of the hands, your ability to move it fast and slow is the same? The improvements that you were talking about earlier are about basically not moving when you don't want it to and moving when you do?

Pilot

Yeah.

Researcher

You were saying earlier that when you supinate, sometimes the hand will open. Is that something that has been around for the entire time that you have had the wrist?

Pilot

Yeah, I think so. Yeah.

Researcher

Would you say that your use of the wrist has increased since the Cybathlon, or has been the same since you had it?

Pilot

Yeah, yeah it has been better.

Researcher

For all of the things that you have with your arm, if you could improve something about it, what would you improve?

Pilot

The fingers. I want to add few fingers.

Researcher

Why do you want to add the finger movements?

Pilot

Silly question! (laughter) So I can take small things like a credit card, everything. It feels more natural. And maybe my phantom pain can disappear if I can move my fingers.

Researcher

On the topic of phantom limb pain, do you feel like your training for the Cybathlon has had any influence on your phantom pain?

Pilot

No.

Researcher

It was the same?

Pilot

Yeah. Maybe more when I trained a lot … right? (He looks questioningly at his wife)

Pilot's Wife

You slept way better when you were here and trained. Then you didn't have pain.

Pilot

True, in the hotel. But I always slept better in hotels.

Researcher

But then since training, it has gone back to the same level it was before?

Pilot

Yeah.

Researcher

Last year, you had come into the lab many times leading up to the competition. Do you feel that training at lab was helpful?

Pilot

Yes.

Researcher

Or was the training at home the main helper?

Pilot

I think both.

Researcher

What was it about the lab training that you found helpful?

Pilot

Maria and Andrew pushed me a lot. My confidence is better. And Jan did some changes in the system.

Researcher

If you have the chance to change something in the way that you trained for the Cybathlon, what would you change?

Pilot

I think nothing.

Researcher

And in what ways do you think that our team can improve for the next Cybathlon?

Pilot

I don't know

Researcher

More Andrews and Marias? (laughter)

Pilot

No, I think you did a good job.

Researcher

Oh, thank you.

Pilot

Maybe more practice with [the “e-OPRA” pilot]. We can help each other.

Researcher

During the actual Cybathlon competition, did participating in the competition match your expectations?

Pilot

Yes, but I thought it would be in Zurich. (laughter).

Researcher

For you, what were the highlights of the entire experience?

Pilot

When [the “e-OPRA” pilot] came on the 3d place.

Researcher

Overall, what do you think is the impact that the Cybathlon has had on your life?

Pilot

My confidence has been much better.

Researcher

The confidence in your control?

Pilot

Yeah, and standing in front of a camera and a lot of people... and I trust my prosthesis more. And I got a new friend.

Researcher

Who is it?

Pilot

[the “e-OPRA” pilot] (laughter).

Researcher

What was the biggest challenge for you participating in the Cybathlon?

Pilot

The camera and the people. But it was not as hard as I thought.

Researcher

And lastly, would you participate in the Cybathlon again if you had the chance?

Pilot

Yes.

Researcher

I think that was everything, thank you.
